# Supplementary material for: Disadvantaged Americans are suffering the brunt of rising pain and physical limitations
Source: PLoS One. 2021 Dec 9;16(12):e0261375. doi: 10.1371/journal.pone.0261375 (PMC8659634; doi:10.1371/journal.pone.0261375)
Supplement: S1 Appendix — (DOCX) [file pone.0261375.s001.docx]

S1 Appendix: Supplementary Material

# Text A. Construction of the SES index

Relative SES was based on the educational attainment of the respondent (and spouse/partner, if applicable), the current or most recent occupation of the respondent (and spouse/partner, if applicable), income, and current net assets of the respondent and spouse (see Table D). A similar measure has been used in several earlier studies (1–7). Unfortunately, a measure of wealth was not available from NHIS; the SES index for that survey was based only on the other three components.

Because income and assets were strongly and positively skewed, we applied a square root transformation to those two items. Then, within each wave for each survey, we standardized all component items and calculated the mean across relevant items (e.g., six items if married/partnered and both respondent and spouse/partner have ever been employed; three items if not married/partnered and respondent has never been employed). The Cronbach’s α ranged from 0.78 to 0.81 in HRS, 0.74-0.75 in MIDUS, and 0.65-0.67 in NHIS.

We also created an alternative SES index for HRS and MIDUS based only on the three items that are comparable with NHIS. The Cronbach’s α ranged from 0.66 to 0.70 in HRS and was 0.64-0.66 in MIDUS.

## A.1 Income and Wealth Measures in HRS

For HRS, we used the total household income constructed by the RAND Center for the Study of Aging (8, pp. 1111-1113). That variable comprised income for the respondent and spouse (but not other members of the household) in the last calendar year. Similarly, we used the RAND-constructed measure of total wealth excluding secondary residence (8, pp. 999-1000). Total wealth including secondary residence could not be computed for the 1996 Wave because the survey did not ask about a second home in that wave.

If an income or wealth component was missing, RAND imputed the value (8, see pp. 22-31). The percentage of respondents for which at least one income component was imputed ranged from 40% in 2016 to 59% in 2002.

## A.2 Income and Wealth Measures in MIDUS

At both waves of MIDUS, income from each source (i.e., wages/salary, social security, government assistance, and all other sources such as pensions, investments, child support, or alimony) was reported in categories. These variables included incomes provided by all family members living in the household. We coded income from each source to the mid-point of the range within each category and then summed across all sources to compute total income. In the 1995-96 wave, income from each source was top-coded at $200,000 (except government assistance, which is top-coded at $50,000); 1.3% of respondents had top-coded income from one or more sources. For the 2011-14 wave, income was top-coded at $300,000; 0.8% of respondents had top-coded income from at least one source. We recoded those top-coded values to the harmonic mean of a Pareto distribution. As suggested by von Hippel (9), we computed the harmonic mean of a Pareto distribution with $\alpha$ equal to the maximum of one or $\frac{\ln\left( n_{B-1}+n_{B} \right)-ln(n_{B})}{\ln\left( l_{B} \right)-ln(l_{B-1})}$, where $n_{B}$ was the number of cases in the top category; $n_{B-1}$ was the number of cases in the penultimate category; $l_{B}$ was the lower bound of the top category; and $l_{B-1}$ is the lower bound of the penultimate category. Restricting alpha to a minimum of one ensures that the value of the top category was no greater than twice the lower bound of that category. We were unable to make an equivalence adjustment based on household size and composition because MIDUS did not collect that information at wave 1.

Assets were also reported in categories in 1995-96, which we coded to the mid-point of each range. In 2011-14, the dollar amount of assets was recorded. If the respondent reported no assets or a deficit, total net assets was coded to zero (i.e., no information is available regardless the magnitude of indebtedness). At both waves, assets were top-coded at $1,000,000 (2.3% of the sample in 1995-96 and 8.2% in 2011-14). We recoded the top-coded values to the harmonic mean of a Pareto distribution as described above for income.

## A.3 Income and Wealth Measures in NHIS

For NHIS, we used the total combined family income from the imputed income file (10). To preserve confidentiality, the data provider top-coded the income variable to the 95^th^ percentile of the appropriate distribution. Given high levels of missing data (i.e., 19-34% were missing family income during the 1997-2018 waves), NHIS used multiple imputation methods to handle missing data on family income. Measures of wealth were not available from NHIS.

# Text B. Missing data and multiple imputation

In HRS, very few observations were missing race/ethnicity (0.1%), SES (<0.01%), or measures of overall pain (i.e., any pain or pain limits activity) (<0.5%). A higher proportion were missing data for one of the physical tasks (<2% missing for stooping or walking several blocks, 3% missing for lifting, and 8% missing for stair climbing), but the vast majority of those cases (83% for stooping, 11% for walking, 93% for lifting, and 96% for stair climbing) were missing because the respondents reported they “don’t do” that activity. The questions about back pain and headaches were not asked in every wave and thus, were missing by design for 42% of observations.

For NHIS, only a few respondents were missing data for SES (0.5% were missing SES), each of the pain measures (0.1%), and each of the physical tasks (1-2%).

For MIDUS, at least one component of income was missing for 10% of respondents in 1995‑96 and 26% in 2011-14. Similarly, information regarding assets was missing for 9% and 18% of respondents in the respective MIDUS waves. The only other analysis variable in MIDUS with substantial missing data (8%) was Latinx ethnicity in 1995-96. Thus, we used the *ice* command in Stata 16.1 (11) to implement multiple imputation for missing data (12,13). The set of predictors for multiple imputation included all the analysis variables as well as several auxiliary variables: self-assessed health status; prevalence of selected chronic conditions (e.g., cancer, heart disease, diabetes, lung problems, arthritis); measures of psychological distress and well-being, exercise frequency, smoking, drug misuse and abuse, alcohol abuse, self-reported weight, height, and waist circumference); and whether the respondent died by the end of follow-up.

For NHIS and MIDUS, we used the “mim” prefix command to re-estimate the model for each of the five multiply-imputed datasets. Then, we combine the five sets of estimates using Rubin’s rules (14).

Table A. Information regarding sampling design, response rates, and analytic samples for each dataset

|  | **HRS 1996-2016** | **MIDUS 1 (1995-96) &**  **MIDUS Refresher (2011-14)** | **NHIS 1997-2018** |
| --- | --- | --- | --- |
| Survey Type | Longitudinal | Cross-sectional | Cross-sectional |
| Population sampled | US, persons born in 1890-1965 (and their spouse/partners) residing in households within the contiguous US | US, non-institutionalized, English speaking residents of the contiguous US | US, civilian, noninstitutionalized residents of all 50 states and the District of Columbia |
| Age range | 18+^a^ | 20-76^f^ | 18+ (Sample Adults) |
| Sampling design | Multi-stage probability  sample with geographical stratification and clustering. | At Wave 1 (1995-96), the main sample was selected by national random digit dialing (15). The Refresher sample (2011-14) was drawn from the national population using a sampling frame that included both landlines and cell phones (16). | Stratified, geographically-clustered probability sample. For the sample adult questionnaire, one adult per family is randomly selected. |
| Subgroup(s) oversampled | Blacks;  Latinx;  Florida residents | In 1995-96:   - Older persons - Men   In 2011-14:   - None | Prior to 2016:   - Blacks - Latinx - Asians   Since 2016:   - None |
| Interviewed | 39,777 (respondents)  215,180 (observations) | 5,632^g^ | 671,969 |
| Survey Mode | Face-to-Face OR  Telephone^b^ | Phone (initial interview) AND  SAQ | Face-to-Face |
| Response Rate | First wave for each cohort:^c^  68.8% (Cohorts entering in 2010) to  81.6% (Cohorts entering in 1992)  Follow-up waves (1996-2014):  86.9% (1996) to  89.1% (2012) | Completed phone interview AND SAQ:  61% (1995-96)  43% (2011-14) | 53.0% (2017) to  80.4% (1997)  (17, see Table II in Appendix II) |
| **Excluded from analysis:** | | | |
| Living in an institution | 4,532 (observations)^d^ | -- | -- |
| Other reasons | Not age-eligible^e^ (*N*=13,397)  Sampling weight is zero or missing (*N*=48) | -- | -- |
| Missing a predictor | 161 | -- | 3088 |
| Missing all outcomes | 9 | -- | 25 |
| **Analysis sample** | **190,546**  (aged 50+) | **5,632**  (aged 20-76) | **668,526**  (aged 18+)^h^ |

Abbreviations: HRS, Health and Retirement Study; MIDUS, Midlife in the United States study; NHANES, National Health and Nutritional Examination Survey; NHIS, National Health Interview Survey; SAQ, Self-Administered Questionnaire.

^a^ In 1992, HRS sampled cohorts born in 1931-41 when they were approximately age 51-61. In subsequent waves, they sampled the cohorts born in 1923 or earlier (first interviewed in 1993), those born in 1924-30 and 1942-47 (first interviewed in 1998), cohorts born in 1948-53 (first interviewed in 2004), those born in 1954-59 (first interviewed in 2010-11), and cohorts born in 1960-65 (first interviewed in 2016). Each cohort is re-interviewed every two years. Thus, the longitudinal sample is representative of the US population older than age 50 in 1998, 2004, 2010, and 2016; at other waves, the age range is more restricted. In addition to the targeted age-eligible respondent, HRS also interviewed the current spouse/partner (regardless of their age) at the time of the initial interview and at the time of all subsequent interviews.

^b^ Most of the baseline interviews for HRS were conducted face-to-face, but subsequent interviews were often administered by telephone (18). Prior to 2004, follow-up interviews were conducted primarily by telephone although respondents over the age of 80 were offered a face-to-face interview. Since 2006, HRS has used a mixed-mode design for follow-up: about half of the interviews are conducted in-person, while the other half are administered via telephone. For supplemental studies, HRS also uses internet and self-administered mail surveys.

^c^ Response rates reflect the percentage who completed the HRS baseline interview among those deemed eligible for HRS (19, see Table 1). To our knowledge, HRS has not published the response rates for the 2016 or 2018 waves.

^d^ At baseline, HRS interviews only respondents living in the community, but during follow-up waves respondents who became institutionalized are retained and interviewed.

^e^ For HRS, we restricted our analyses to the cohorts sampled (e.g., individuals born in 1960-65 were not age-eligible until the 2016 wave).

^f^ Although MIDUS targeted persons aged 25-74, a few of the eventual respondents were outside that age range at the time of the initial phone interview: at the 1995-96 wave, there was *N=*1 respondent aged 20 and *N=*9 aged 24, but none were older than 74; at the 2011-14 wave, there were *N=*2 respondents aged 23, *N=*3 aged 24, *N=*57 aged 75, and *N=*1 aged 76.

^g^ Respondents who completed both the phone interview and self-administered questionnaire (SAQ).

^h^ Age was top-coded at 85+ in NHIS.

Table B. Wording of the physical limitation questions by survey

| **Survey** | **Introductory text** | **Response categories^a^** | **Walking**  **a short**  **distance** | **Lifting/**  **carrying** | **Climbing**  **stairs** | **Bending/**  **stooping/**  **kneeling, etc.** |
| --- | --- | --- | --- | --- | --- | --- |
| HRS | *Please tell me whether you have any difficulty doing each of the everyday activities that I read to you. Exclude any difficulties that you expect to last less than three months. Because of a health problem do you have any difficulty with...?* | *0) No*  *1) Yes*  *2) Can’t do*  *9) Don’t do* | *Walking several blocks*^b^ | *Lifting or carrying weights over 10 pounds, like a heavy bag of groceries* | *Climbing several flights of stairs without resting* | *Stooping, kneeling, or crouching* |
| MIDUS | *How much does your health limit you in doing each of the following?* [no response category for “*do not do this activity*”] | *0) Not at all*  *1) A little*  *2) Some*  *3) A lot* | *Walking several blocks*^b^ | *Lifting or carrying groceries*^c^ | *Climbing several flights of stairs* | *Bending,*  *kneeling,*  *or stooping^d^* |
| NHIS | *By yourself, and without using any special equipment, how difficult is it for you to...?* | *0) Not at all difficult*  *1) Only a little difficulty*  *2) Somewhat difficult*  *3) Very difficult*  *4) Can’t do it at all*  *9) Do not do this activity* | *Walk a quarter of a mile* *- about 3 city blocks* | *Lift or carry something as heavy as 10 pounds* *such as a full bag of groceries?* | *Walk up 10 steps without resting* | *Stoop, bend, or kneel^d^* |

^a^ We classified responses denoted by the code 0 (e.g., “No”, “Not at all”) as indicating no difficulty and the remaining responses (e.g., “yes”, “can’t do”, “a little”, “some”, “a lot”) denoted by codes 1-4 as indicating difficulty. Responses shown in red (e.g., “Don’t do”, “Do not do this activity”) were coded as missing. MIDUS did not include a “Do not do it” category, so there is no way to know how such respondents answered the question.

^b^ Depends on the respondent’s interpretation of the question. Short blocks are typically 100m long, while long blocks are generally 200m long (i.e., ≈1/8 of a mile). So, if the respondent interpreted “several blocks” to mean three short blocks that would be 300m (i.e., shorter than 1/4 of a mile), whereas if s/he interpreted it to mean three long blocks it would be 600m (i.e., longer than 1/4 of a mile).

^c^ Plastic limited-use grocery bags are designed to hold up to 17 lbs.

^d^ Although the defined physical activities were virtually identical in these surveys, the ordering of the tasks differs (i.e., MIDUS mentions “bending” first, whereas HRS and NHIS mentions “stoop(ing)” first).

Table C. Wording of the pain questions by survey

| **Survey** | **Question(s)** | **Response categories** |
| --- | --- | --- |
| HRS | Are you often troubled by pain? | No  Yes |
|  | Does the pain make it difficult for you to do your usual activities such as household chores or work? | No  Yes |
|  | ([*Re-Interviews*] Since we last talked to you,) Have you had any of the following persistent or troublesome problems? a) back pain or problems; b) persistent headaches?^a^ | No  Yes |
| MIDUS | During the past 30 days, how often have you experienced each of the following:  a) headaches; b) lower backaches; c) aches or stiffness in joints | Not at all  Once a month  Several times a month  Once a week  Several times a week  Almost every day |
| NHIS | During the past 3 months, did you have: a) Neck pain; b) Low back pain; c) facial ache or pain in the jaw muscles or the joint in front of the ear; d) severe headache or migraine? | No  Yes |
|  | [Since the 2002 Wave only] During the past 30 days, have you had any symptoms of pain, aching, or stiffness in or around a joint? | No  Yes |

^a^ Only new respondents were asked this question dents in 1998, 2002, 2006, 2010, 2014, and 2018.

Table D. Components of the SES index by survey

| **Component** | **Survey** | **Question(s)** | **Coding** |
| --- | --- | --- | --- |
| Respondent’s education | HRS | What is the highest grade of school or year of college you completed? | 1=No degree (i.e., less than a GED)  2=HS graduate/GED  3=Some college (including Associate’s degree)  4=Bachelor’s degree  5=Master’s degree  6=PhD/Law/MD or other professional degree |
|  | MIDUS | What is the highest grade of school or year of college you completed? *(from phone interview)^a^* |  |
|  | NHIS | What is the highest level of school {person} has completed or the highest degree {person has} received? *(from the Family Core component*)^a^ |  |
| S/P’s education | HRS | *(Same as for Respondent’s education)* | *(Same as for Respondent’s education)* |
|  | MIDUS | What is the highest grade of school or year of college your (spouse/partner) completed? *(from phone interview)^a^* |  |
|  | NHIS | N/A |  |
| Respondent’s occupation | HRS | The RAND occupational variables [**R*JCOCC***] were based on the 1980 census occupation codes for the 1992-2002 HRS, the 2000 census occupation codes for the 2004-08 waves, and the 2010 census occupational codes for the 2010-18 waves. | 1=Farming/Construction/Maintenance/  Production/Transportation/Military  2=Service/Sales/Administration/Office  3=Management/Business/Financial  4=Professional |
|  | MIDUS | Occupation was coded based on the 1980 census occupation codes in 1995-96 and the 2010 census occupation codes in 2011-14. |  |
|  | NHIS | Occupation was coded based on the 1995 SOC codes prior to 2004, the 2000 SOC codes for the 2004-09 waves, and the 2010 SOC codes since 2010. |  |
| S/P’s occupation | HRS | *(Same as for Respondent’s occupation)* | *(Same as for Respondent’s occupation)* |
|  | MIDUS |  |  |
|  | NHIS | N/A |  |
| Income | HRS | Annual income for the respondent and S/P | Values were top-coded in MIDUS and NHIS (see Section A.1-A.3 for details). |
|  | MIDUS | Annual household income was calculated including the respondent, spouse/partner (if applicable), and all other family members living in the household |  |
|  | NHIS | Total combined family income |  |
| Wealth | HRS | Total net assets (excluding second home) for the respondent and S/P combined. | We bottom-coded assets in order to make it  more comparable with the measure in MIDUS. |
|  | MIDUS | Total net assets for the respondent and S/P combined. | Assets were bottom-coded at $0. |
|  | NHIS | N/A |  |

Abbreviations: N/A, Not applicable; S/P, Spouse/partner.

^a^ The question in MIDUS included 12 response categories ranging from “No school/some grade school” to “PhD, EdD, MD, DDS, LLB, LLD, JD, or other professional degree.” The question in NHIS included 22 response categories ranging from “never attended/kindergarten only” to “doctoral degree (example: PhD, EdD).” To maximize comparability with HRS, we recoded the responses from MIDUS and NHIS into the six categories available for HRS.

Table E. Descriptive statistics for analysis variables by survey, weighted analyses

| **Variable** | **HRS**  **1996-2018** | **MIDUS**  **1995-96 & 2011-14** | **NHIS**  **1997-2018** |
| --- | --- | --- | --- |
| Female, % | 54.3 | 52.2 | 51.8 |
| Age in years, mean (SD) | 65.5 (10.1) | 47.1 (13.6) | 46.1 (17.7) |
| Non-Latinx White, % | 79.3 | 81.0 | 69.5 |
| Non-Latinx Black, % | 9.6 | 8.0 | 11.7 |
| Non-Latinx other race, % | 3.2 | 5.4 | 4.9 |
| Latinx, % | 7.9 | 5.6 | 14.0 |
| Relative SES (1-100),^a^ mean (SD) | 50.5 (28.9) | 50.5 (28.9) | 50.5 (28.9) |
| Any physical limitation,^b^ % | 56.3 | 53.0 | 26.9 |
| Limitation lifting/carrying,^b^ % | 21.2 | 27.8 | 10.3 |
| Limitation climbing stairs,^b^ % | 41.6 | 36.9 | 12.2 |
| Limitation kneeling/stooping,^b^ % | 42.8 | 43.1 | 23.7 |
| Limitation walking,^b^ % | 27.2 | 27.9 | 15.8 |
| Often troubled by pain,^b^ % | 33.9 | N/A | N/A |
| Pain limits activity,^b^ % | 21.7 | N/A | N/A |
| Headache,^b^ % | 10.0 | 23.3 | 15.1 |
| Facial/jaw pain,^b^ % | N/A | N/A | 4.6 |
| Neck pain,^b^ % | N/A | N/A | 14.9 |
| Back pain,^b^ % | 37.5 | 28.4 | 28.2 |
| Joint pain,^c^ % | N/A | 38.5 | 32.1 |
| Number of observations | 190,546 | 5,632 | 668,526 |
| Number of unique respondents | 36,535 | 5,632 | 668,526 |

N/A, Not Available.

^a^ SES was based on education, occupation, income, and assets; the index scores were converted to a percentile denoting the respondents’ rank within the distribution at the specified survey wave (1=bottom percentile, 100=top percentile).

^b^ There were some missing values for outcomes in NHIS (0.1% for pain measures; 1-2% for individual physical limitations; 3.4% for any physical limitation) and HRS (0.2-0.3% missing often troubled by pain and pain limits activity; 42% missing by design for headache and back pain, which were asked only of new respondents; 1-8% missing for individual physical limitations, 83-96% of which say they “do not do it”; 11% missing for any physical limitation).

^c^ Joint pain was available from NHIS only since 2002 (missing for 0.1%, leaving *N*=504,194).

Table F. Coefficients (and 95% CIs) from logistic regression models predicting physical limitation outcomes, 1996-2016 HRS

|  | **Any**  **Limitation** | **Limitation**  **Lifting/**  **Carrying** | **Limitation**  **Climbing**  **Stairs** | **Limitation**  **Kneeling/**  **Stooping** | **Limitation**  **Walking** |
| --- | --- | --- | --- | --- | --- |
| Female | 1.18*** | 1.54*** | 1.21*** | 0.72*** | 0.65*** |
|  | (1.11 - 1.25) | (1.48 - 1.61) | (1.15 - 1.27) | (0.65 - 0.78) | (0.58 - 0.72) |
| Age – 50 | 0.22 | 0.10 | 0.06 | 0.11 | 0.06 |
|  | (-0.05 - 0.49) | (-0.15 - 0.34) | (-0.19 - 0.31) | (-0.12 - 0.33) | (-0.18 - 0.30) |
| (Age – 50)^2^ | 0.07 | 0.09** | 0.06* | 0.04 | 0.15*** |
|  | (-0.00 - 0.13) | (0.03 - 0.15) | (0.00 - 0.12) | (-0.02 - 0.09) | (0.09 - 0.21) |
| Non-Latinx White | *omitted* | *omitted* | *omitted* | *omitted* | *omitted* |
| Non-Latinx Black | 0.04 | 0.66*** | 0.25*** | -0.16** | 0.34*** |
|  | (-0.08 - 0.15) | (0.54 - 0.77) | (0.13 - 0.36) | (-0.26 - -0.07) | (0.22 - 0.46) |
| Non-Latinx Other Race | -0.13 | 0.57*** | 0.07 | -0.30** | 0.18 |
|  | (-0.36 - 0.11) | (0.34 - 0.79) | (-0.14 - 0.28) | (-0.51 - -0.10) | (-0.07 - 0.42) |
| Latinx | -0.39*** | 0.04 | -0.17* | -0.42*** | -0.47*** |
|  | (-0.55 - -0.23) | (-0.11 - 0.20) | (-0.31 - -0.03) | (-0.56 - -0.28) | (-0.64 - -0.29) |
| Relative SES^a^ | -3.10*** | -3.20*** | -3.72*** | -2.79*** | -4.44*** |
|  | (-3.36 - -2.84) | (-3.52 - -2.88) | (-3.99 - -3.44) | (-3.02 - -2.56) | (-4.80 - -4.08) |
| SES x (Age – 50) | 0.54*** | 0.03 | 0.65*** | 0.70*** | 0.46** |
|  | (0.28 - 0.80) | (-0.25 - 0.32) | (0.39 - 0.90) | (0.47 - 0.93) | (0.13 - 0.79) |
| SES x (Age – 50)^2^ | 0.04 | 0.16*** | 0.07* | -0.01 | 0.09* |
|  | (-0.03 - 0.10) | (0.10 - 0.23) | (0.00 - 0.13) | (-0.06 - 0.04) | (0.01 - 0.17) |
| 1996-99 | *omitted* | *omitted* | *omitted* | *omitted* | *omitted* |
| 2000-03 | 0.09 | 0.09 | 0.19 | 0.28** | 0.22* |
|  | (-0.13 - 0.30) | (-0.14 - 0.32) | (-0.02 - 0.39) | (0.08 - 0.47) | (0.01 - 0.43) |
| 2004-07 | 0.20 | 0.11 | 0.10 | 0.26* | 0.03 |
|  | (-0.05 - 0.45) | (-0.14 - 0.35) | (-0.15 - 0.35) | (0.06 - 0.47) | (-0.21 - 0.26) |
| 2008-11 | 0.17 | 0.40** | -0.02 | 0.33** | 0.10 |
|  | (-0.09 - 0.42) | (0.13 - 0.68) | (-0.25 - 0.21) | (0.09 - 0.57) | (-0.12 - 0.32) |
| 2012-15 | 0.29* | 0.53*** | 0.20 | 0.37** | 0.39** |
|  | (0.02 - 0.56) | (0.24 - 0.81) | (-0.05 - 0.45) | (0.11 - 0.62) | (0.14 - 0.64) |
| 2016-19 | 0.34* | 0.71*** | 0.17 | 0.56*** | 0.66*** |
|  | (0.06 - 0.61) | (0.43 - 1.00) | (-0.08 - 0.42) | (0.30 - 0.82) | (0.38 - 0.94) |
| (Age – 50) x |  |  |  |  |  |
| 2000-03 | 0.14 | -0.00 | 0.01 | 0.00 | -0.02 |
|  | (-0.09 - 0.37) | (-0.23 - 0.23) | (-0.19 - 0.21) | (-0.19 - 0.19) | (-0.24 - 0.20) |
| 2004-07 | 0.26 | -0.08 | 0.30* | 0.16 | 0.22 |
|  | (-0.01 - 0.54) | (-0.34 - 0.17) | (0.03 - 0.58) | (-0.06 - 0.37) | (-0.02 - 0.46) |
| 2008-11 | 0.37* | -0.22 | 0.31* | 0.20 | 0.16 |
|  | (0.09 - 0.65) | (-0.52 - 0.09) | (0.04 - 0.57) | (-0.04 - 0.43) | (-0.08 - 0.41) |
| 2012-15 | 0.14 | -0.29 | 0.15 | 0.22 | -0.01 |
|  | (-0.13 - 0.42) | (-0.60 - 0.03) | (-0.11 - 0.40) | (-0.02 - 0.45) | (-0.28 - 0.26) |
| 2016-19 | 0.17 | -0.33* | 0.03 | 0.03 | -0.29 |
|  | (-0.11 - 0.44) | (-0.64 - -0.02) | (-0.25 - 0.30) | (-0.22 - 0.28) | (-0.59 - 0.01) |
| (Age – 50)^2^ x |  |  |  |  |  |
| 2000-03 | -0.02 | 0.01 | 0.01 | -0.01 | 0.02 |
|  | (-0.09 - 0.04) | (-0.05 - 0.06) | (-0.04 - 0.06) | (-0.06 - 0.04) | (-0.03 - 0.08) |
| 2004-07 | -0.01 | 0.06 | -0.03 | -0.02 | 0.02 |
|  | (-0.08 - 0.06) | (-0.01 - 0.12) | (-0.09 - 0.04) | (-0.07 - 0.04) | (-0.04 - 0.08) |
| 2008-11 | -0.02 | 0.11** | -0.00 | -0.02 | 0.05 |
|  | (-0.09 - 0.06) | (0.03 - 0.18) | (-0.07 - 0.07) | (-0.08 - 0.04) | (-0.02 - 0.11) |
| 2012-15 | 0.05 | 0.14*** | 0.04 | -0.02 | 0.09** |
|  | (-0.02 - 0.13) | (0.06 - 0.21) | (-0.02 - 0.11) | (-0.07 - 0.04) | (0.02 - 0.16) |
| 2016-19 | 0.04 | 0.14*** | 0.09* | 0.03 | 0.16*** |
|  | (-0.03 - 0.12) | (0.06 - 0.22) | (0.02 - 0.15) | (-0.03 - 0.09) | (0.08 - 0.23) |
| SES x |  |  |  |  |  |
| 2000-03 | -0.13 | -0.27** | -0.09 | -0.06 | 0.05 |
|  | (-0.32 - 0.05) | (-0.47 - -0.07) | (-0.31 - 0.13) | (-0.22 - 0.09) | (-0.15 - 0.26) |
| 2004-07 | -0.37*** | -0.34** | -0.36** | -0.12 | -0.14 |
|  | (-0.58 - -0.16) | (-0.58 - -0.11) | (-0.57 - -0.14) | (-0.31 - 0.07) | (-0.40 - 0.11) |
| 2008-11 | -0.61*** | -0.60*** | -0.39*** | -0.26* | -0.30* |
|  | (-0.82 - -0.39) | (-0.88 - -0.33) | (-0.61 - -0.18) | (-0.49 - -0.02) | (-0.59 - -0.02) |
| 2012-15 | -0.82*** | -0.79*** | -0.64*** | -0.59*** | -0.59*** |
|  | (-1.08 - -0.56) | (-1.05 - -0.53) | (-0.88 - -0.39) | (-0.84 - -0.34) | (-0.89 - -0.29) |
| 2016-19 | -1.11*** | -1.22*** | -0.75*** | -0.77*** | -0.74*** |
|  | (-1.37 - -0.85) | (-1.55 - -0.89) | (-1.01 - -0.48) | (-1.02 - -0.52) | (-1.04 - -0.43) |
| Constant | 0.48*** | -2.66*** | -0.44*** | -0.54*** | -1.71*** |
|  | (0.23 - 0.73) | (-2.90 - -2.43) | (-0.66 - -0.21) | (-0.75 - -0.32) | (-1.93 - -1.48) |
| Observations | 169,837 | 184,940 | 176,141 | 187,905 | 187,201 |
| Number of Respondents | 35,448 | 36,179 | 35,841 | 36,397 | 36,335 |
| Rho (ρ)^b^ | 0.69 | 0.65 | 0.68 | 0.63 | 0.74 |

Note: The interactions between period and age (quadratic specification) were jointly significant (p<0.05) for all outcomes.

* p<0.05; ** p<0.01; *** p<0.001

^a^ Rescaled from 0 (bottom percentile) to 1 (top percentile). Because SES was interacted with age and period, the main effect of SES represents the odds ratio for a person aged 50 in the top 1% relative to the bottom 1% of the SES in 1996-99.

^b^ Rho (ρ) indicates the proportion of the total variance that is contributed by the individual-level variance component (i.e., within-individual correlation coefficient).

Table G. Coefficients (and 95% CIs) from logistic regression models predicting pain outcomes, 1996-2016 HRS

|  | **Often Troubled**  **by Pain** | **Pain Limits**  **Activity** | **Headache** | **Back Pain** |
| --- | --- | --- | --- | --- |
| Female | 0.53*** | 0.67*** | 1.13*** | 0.34*** |
|  | (0.47 - 0.59) | (0.60 - 0.74) | (1.02 - 1.24) | (0.28 - 0.40) |
| Age – 50 | -0.61*** | -0.72*** | -1.46*** | -1.39*** |
|  | (-0.82 - -0.40) | (-0.94 - -0.51) | (-1.97 - -0.94) | (-1.77 - -1.01) |
| (Age – 50)^2^ | 0.10*** | 0.10*** | 0.38*** | 0.44*** |
|  | (0.05 - 0.15) | (0.04 - 0.15) | (0.20 - 0.57) | (0.30 - 0.58) |
| Non-Latinx White | *omitted* | *omitted* | *omitted* | *omitted* |
| Non-Latinx Black | -0.40*** | -0.33*** | -0.03 | -0.34*** |
|  | (-0.49 - -0.30) | (-0.44 - -0.21) | (-0.17 - 0.10) | (-0.43 - -0.24) |
| Non-Latinx Other Race | -0.00 | 0.08 | 0.35** | -0.02 |
|  | (-0.21 - 0.21) | (-0.13 - 0.28) | (0.11 - 0.59) | (-0.20 - 0.15) |
| Latinx | -0.36*** | -0.51*** | 0.33*** | -0.56*** |
|  | (-0.49 - -0.23) | (-0.67 - -0.35) | (0.17 - 0.50) | (-0.67 - -0.45) |
| Relative SES^a^ | -2.72*** | -3.42*** | -1.77*** | -1.81*** |
|  | (-2.97 - -2.47) | (-3.73 - -3.11) | (-2.16 - -1.38) | (-2.06 - -1.56) |
| SES x (Age – 50) | 0.80*** | 0.72*** | -0.45 | 0.10 |
|  | (0.54 - 1.06) | (0.43 - 1.01) | (-0.90 - 0.01) | (-0.17 - 0.37) |
| SES x (Age – 50)^2^ | -0.06 | 0.01 | 0.11 | 0.12*** |
|  | (-0.13 - 0.00) | (-0.06 - 0.08) | (-0.01 - 0.23) | (0.06 - 0.18) |
| 1996-99 | *omitted* | *omitted* | *omitted* | *omitted* |
| 2000-03 | 0.30** | 0.18 | -0.34 | -0.30* |
|  | (0.08 - 0.52) | (-0.07 - 0.44) | (-0.71 - 0.03) | (-0.57 - -0.04) |
| 2004-07 | 0.41*** | 0.29** | -0.06 | 0.00 |
|  | (0.21 - 0.60) | (0.09 - 0.49) | (-0.44 - 0.32) | (-0.25 - 0.25) |
| 2008-11 | 0.66*** | 0.57*** | -0.18 | 0.25 |
|  | (0.44 - 0.88) | (0.31 - 0.82) | (-0.55 - 0.19) | (-0.01 - 0.52) |
| 2012-15 | 0.86*** | 0.80*** | -0.49* | -0.10 |
|  | (0.63 - 1.09) | (0.54 - 1.07) | (-0.89 - -0.08) | (-0.38 - 0.17) |
| 2016-19 | 1.21*** | 1.07*** | 0.02 | 0.47*** |
|  | (0.98 - 1.45) | (0.80 - 1.35) | (-0.39 - 0.43) | (0.19 - 0.74) |
| Interactions: (Age – 50) x |  |  |  |  |
| 2000-03 | 0.01 | 0.07 | 0.75** | 0.93*** |
|  | (-0.19 - 0.21) | (-0.17 - 0.31) | (0.26 - 1.25) | (0.57 - 1.30) |
| 2004-07 | 0.21* | 0.17 | 0.51* | 0.92*** |
|  | (0.02 - 0.39) | (-0.01 - 0.36) | (0.01 - 1.00) | (0.54 - 1.29) |
| 2008-11 | 0.23* | 0.19 | 0.46 | 0.89*** |
|  | (0.01 - 0.45) | (-0.07 - 0.46) | (-0.08 - 1.00) | (0.52 - 1.25) |
| 2012-15 | 0.18 | 0.14 | 1.09*** | 1.38*** |
|  | (-0.03 - 0.39) | (-0.10 - 0.39) | (0.55 - 1.62) | (1.00 - 1.76) |
| 2016-19 | 0.12 | 0.11 | 0.59* | 1.14*** |
|  | (-0.11 - 0.34) | (-0.14 - 0.35) | (0.06 - 1.12) | (0.75 - 1.53) |
| Interactions: (Age – 50)^2^ x |  |  |  |  |
| 2000-03 | -0.01 | -0.02 | -0.34*** | -0.40*** |
|  | (-0.06 - 0.03) | (-0.08 - 0.03) | (-0.52 - -0.16) | (-0.54 - -0.27) |
| 2004-07 | -0.06* | -0.03 | -0.29** | -0.41*** |
|  | (-0.10 - -0.01) | (-0.08 - 0.01) | (-0.47 - -0.11) | (-0.55 - -0.28) |
| 2008-11 | -0.06* | -0.04 | -0.28** | -0.41*** |
|  | (-0.11 - -0.00) | (-0.11 - 0.02) | (-0.48 - -0.09) | (-0.55 - -0.28) |
| 2012-15 | -0.04 | -0.03 | -0.43*** | -0.50*** |
|  | (-0.09 - 0.01) | (-0.09 - 0.03) | (-0.61 - -0.24) | (-0.64 - -0.36) |
| 2016-19 | -0.03 | -0.01 | -0.32*** | -0.46*** |
|  | (-0.08 - 0.03) | (-0.07 - 0.05) | (-0.51 - -0.13) | (-0.60 - -0.32) |
| Interactions: SES x |  |  |  |  |
| 2000-03 | -0.07 | -0.13 | 0.08 | -0.06 |
|  | (-0.27 - 0.12) | (-0.35 - 0.10) | (-0.34 - 0.49) | (-0.27 - 0.16) |
| 2004-07 | 0.07 | 0.01 | 0.04 | -0.04 |
|  | (-0.13 - 0.26) | (-0.22 - 0.24) | (-0.34 - 0.43) | (-0.28 - 0.20) |
| 2008-11 | -0.20* | -0.16 | 0.11 | -0.32** |
|  | (-0.38 - -0.02) | (-0.40 - 0.08) | (-0.29 - 0.51) | (-0.54 - -0.09) |
| 2012-15 | -0.27* | -0.35* | -0.37 | -0.37** |
|  | (-0.48 - -0.06) | (-0.62 - -0.08) | (-0.81 - 0.07) | (-0.61 - -0.13) |
| 2016-19 | -0.37** | -0.53*** | -0.19 | -0.50*** |
|  | (-0.62 - -0.12) | (-0.81 - -0.25) | (-0.64 - 0.26) | (-0.76 - -0.24) |
| Constant | -0.56*** | -1.29*** | -2.40*** | 0.29* |
|  | (-0.76 - -0.37) | (-1.51 - -1.07) | (-2.78 - -2.01) | (0.06 - 0.53) |
| Observations | 190,251 | 189,987 | 109,758 | 109,712 |
| Number of Respondents | 36,516 | 36,509 | 35,231 | 35,233 |
| Rho (ρ)^b^ | 0.63 | 0.66 | 0.62 | 0.57 |

Note: The interactions between period and age (quadratic specification) were jointly significant (p<0.05) only for headaches and back pain.

* p<0.05; ** p<0.01; *** p<0.001

^a^ Rescaled from 0 (bottom percentile) to 1 (top percentile). Because SES was interacted with age and period, the main effect of SES represents the odds ratio for a person aged 50 in the top 1% relative to the bottom 1% of the SES in 1996-99.

^b^ Rho (ρ) indicates the proportion of the total variance that is contributed by the individual-level variance component (i.e., within-individual correlation coefficient).

Table H. Coefficients (and 95% CIs) from logistic regression models predicting physical limitation outcomes, 1995-96 and 2011-14 MIDUS

|  | **Any**  **Limitation** | **Limitation**  **Lifting/**  **Carrying** | **Limitation**  **Climbing**  **Stairs** | **Limitation**  **Kneeling/**  **Stooping** | **Limitation**  **Walking** |
| --- | --- | --- | --- | --- | --- |
| Female | 0.47*** | 0.74*** | 0.58*** | 0.31*** | 0.52*** |
|  | (0.34 - 0.60) | (0.58 - 0.89) | (0.44 - 0.71) | (0.18 - 0.45) | (0.36 - 0.67) |
| Age – 50 | 0.37*** | 0.34*** | 0.30*** | 0.33*** | 0.29*** |
|  | (0.26 - 0.49) | (0.22 - 0.46) | (0.19 - 0.41) | (0.22 - 0.44) | (0.17 - 0.40) |
| (Age – 50)^2^ | -0.05 | -0.06 | -0.09* | -0.02 | -0.11** |
|  | (-0.14 - 0.03) | (-0.15 - 0.03) | (-0.18 - -0.01) | (-0.10 - 0.06) | (-0.19 - -0.03) |
| Non-Latinx White | *omitted* | *omitted* | *omitted* | *omitted* | *omitted* |
| Non-Latinx Black | 0.08 | 0.32* | 0.15 | 0.08 | 0.47** |
|  | (-0.20 - 0.35) | (0.02 - 0.62) | (-0.12 - 0.43) | (-0.20 - 0.36) | (0.17 - 0.77) |
| Non-Latinx Other Race | 0.09 | 0.51** | 0.16 | -0.01 | 0.19 |
|  | (-0.22 - 0.39) | (0.19 - 0.82) | (-0.17 - 0.48) | (-0.31 - 0.30) | (-0.15 - 0.54) |
| Latinx | 0.03 | 0.18 | -0.07 | 0.05 | 0.12 |
|  | (-0.25 - 0.32) | (-0.15 - 0.52) | (-0.38 - 0.23) | (-0.25 - 0.35) | (-0.20 - 0.44) |
| Relative SES^a^ | -1.47*** | -1.49*** | -1.82*** | -1.20*** | -2.33*** |
|  | (-1.84 - -1.10) | (-1.90 - -1.08) | (-2.21 - -1.44) | (-1.57 - -0.83) | (-2.76 - -1.90) |
| SES x (Age – 50) | 0.15 | 0.16 | 0.14 | 0.25** | 0.18 |
|  | (-0.03 - 0.34) | (-0.05 - 0.36) | (-0.04 - 0.33) | (0.06 - 0.43) | (-0.02 - 0.38) |
| SES x (Age – 50)^2^ | 0.09 | 0.05 | 0.14* | -0.04 | 0.18* |
|  | (-0.05 - 0.23) | (-0.09 - 0.20) | (0.00 - 0.28) | (-0.18 - 0.10) | (0.04 - 0.32) |
| 1995-96 | *omitted* | *omitted* | *omitted* | *omitted* | *omitted* |
| 2011-14 | 0.59** | 0.61*** | 0.47** | 0.62*** | 0.67*** |
|  | (0.24 - 0.95) | (0.26 - 0.96) | (0.13 - 0.80) | (0.28 - 0.97) | (0.33 - 1.02) |
| (Age – 50) x (2011-14) | 0.03 | -0.00 | 0.06 | 0.02 | 0.07 |
|  | (-0.08 - 0.13) | (-0.12 - 0.11) | (-0.05 - 0.16) | (-0.08 - 0.12) | (-0.05 - 0.18) |
| (Age – 50)^2^ x (2011-14) | -0.00 | -0.04 | 0.03 | -0.01 | -0.00 |
|  | (-0.08 - 0.07) | (-0.12 - 0.05) | (-0.05 - 0.10) | (-0.08 - 0.07) | (-0.09 - 0.08) |
| SES x (2011-14) | -0.66** | -0.80** | -0.64* | -0.57* | -0.93*** |
|  | (-1.15 - -0.17) | (-1.33 - -0.26) | (-1.14 - -0.15) | (-1.06 - -0.09) | (-1.46 - -0.39) |
| Constant | 0.66*** | -0.70*** | 0.05 | 0.16 | -0.23 |
|  | (0.41 - 0.92) | (-0.96 - -0.43) | (-0.20 - 0.30) | (-0.08 - 0.41) | (-0.49 - 0.03) |
| Number of Respondents | 5,632 | 5,632 | 5,632 | 5,632 | 5,632 |

Note: The interactions between period and age (quadratic specification) were not jointly significant for any of these outcomes.

* p<0.05; ** p<0.01; *** p<0.001

^a^ Rescaled from 0 (bottom percentile) to 1 (top percentile). Because SES was interacted with age and period, the main effect of SES represents the odds ratio for a person aged 50 in the top 1% relative to the bottom 1% of the SES in 1995-96.

Table I. Coefficients (and 95% CIs) from logistic regression models predicting pain outcomes, 1995-96 and 2011-14 MIDUS

|  | **Headaches** | **Lower Backaches** | **Joint Aches/Stiffness** |
| --- | --- | --- | --- |
| Female | 0.50*** | 0.04 | 0.04 |
|  | (0.34 - 0.66) | (-0.10 - 0.18) | (-0.10 - 0.17) |
| Age – 50 | -0.32*** | -0.04 | 0.19*** |
|  | (-0.45 - -0.18) | (-0.16 - 0.07) | (0.08 - 0.31) |
| (Age – 50)^2^ | -0.09 | -0.07 | -0.07 |
|  | (-0.19 - 0.01) | (-0.16 - 0.01) | (-0.16 - 0.01) |
| Non-Latinx White | *omitted* | *omitted* | *omitted* |
| Non-Latinx Black | -0.33 | -0.57*** | -0.49*** |
|  | (-0.69 - 0.03) | (-0.91 - -0.24) | (-0.78 - -0.20) |
| Non-Latinx Other Race | -0.21 | -0.17 | -0.13 |
|  | (-0.58 - 0.16) | (-0.48 - 0.15) | (-0.44 - 0.18) |
| Latinx | -0.27 | -0.00 | -0.05 |
|  | (-0.60 - 0.06) | (-0.33 - 0.32) | (-0.36 - 0.27) |
| Relative SES^a^ | -0.72** | -1.03*** | -0.83*** |
|  | (-1.17 - -0.28) | (-1.45 - -0.60) | (-1.20 - -0.45) |
| SES x (Age – 50) | -0.04 | 0.13 | 0.24** |
|  | (-0.26 - 0.18) | (-0.05 - 0.31) | (0.06 - 0.42) |
| SES x (Age – 50)^2^ | 0.09 | 0.12 | 0.07 |
|  | (-0.07 - 0.25) | (-0.02 - 0.26) | (-0.06 - 0.21) |
| 1995-96 | *omitted* | *omitted* | *omitted* |
| 2011-14 | 0.52** | 0.98*** | 1.32*** |
|  | (0.14 - 0.90) | (0.64 - 1.32) | (0.98 - 1.65) |
| (Age – 50) x (2011-14) | 0.08 | -0.02 | 0.00 |
|  | (-0.05 - 0.20) | (-0.12 - 0.09) | (-0.10 - 0.10) |
| (Age – 50)^2^ x (2011-14) | 0.01 | 0.00 | -0.11** |
|  | (-0.08 - 0.10) | (-0.08 - 0.08) | (-0.18 - -0.03) |
| SES x (2011-14) | -0.65* | -0.78** | -0.78** |
|  | (-1.21 - -0.08) | (-1.29 - -0.28) | (-1.25 - -0.31) |
| Constant | -1.21*** | -0.66*** | -0.25 |
|  | (-1.51 - -0.91) | (-0.93 - -0.39) | (-0.50 - 0.00) |
| Number of Respondents | 5,632 | 5,632 | 5,632 |

Note: The interactions between period and age (quadratic specification) were jointly significant (p<0.05) only for joint pain.

* p<0.05; ** p<0.01; *** p<0.001

^a^ Rescaled from 0 (bottom percentile) to 1 (top percentile). Because SES was interacted with age and period, the main effect of SES represents the odds ratio for a person aged 50 in the top 1% relative to the bottom 1% of the SES in 19965-96.

Table J. Coefficients (and 95% CIs) from logistic regression models predicting physical limitation outcomes, 1997-2018 NHIS

|  | **Any**  **Limitation** | **Limitation**  **Lifting/**  **Carrying** | **Limitation**  **Climbing**  **Stairs** | **Limitation**  **Kneeling/**  **Stooping** | **Limitation**  **Walking** |
| --- | --- | --- | --- | --- | --- |
| Female | 0.48*** | 0.93*** | 0.64*** | 0.39*** | 0.52*** |
|  | (0.47 - 0.50) | (0.91 - 0.96) | (0.62 - 0.66) | (0.37 - 0.40) | (0.50 - 0.54) |
| Age – 50 | 0.46*** | 0.47*** | 0.51*** | 0.42*** | 0.49*** |
|  | (0.44 - 0.47) | (0.45 - 0.49) | (0.49 - 0.53) | (0.41 - 0.44) | (0.47 - 0.51) |
| (Age – 50)^2^ | -0.04*** | -0.08*** | -0.07*** | -0.05*** | -0.05*** |
|  | (-0.05 - -0.03) | (-0.09 - -0.07) | (-0.08 - -0.06) | (-0.06 - -0.05) | (-0.06 - -0.05) |
| Non-Latinx White | *omitted* | *omitted* | *omitted* | *omitted* | *omitted* |
| Non-Latinx Black | -0.06*** | 0.29*** | 0.36*** | -0.11*** | 0.15*** |
|  | (-0.08 - -0.03) | (0.26 - 0.32) | (0.33 - 0.39) | (-0.14 - -0.09) | (0.12 - 0.18) |
| Non-Latinx Other Race | -0.61*** | -0.02 | -0.43*** | -0.70*** | -0.50*** |
|  | (-0.66 - -0.57) | (-0.08 - 0.04) | (-0.49 - -0.37) | (-0.75 - -0.65) | (-0.56 - -0.45) |
| Latinx | -0.48*** | -0.14*** | -0.23*** | -0.51*** | -0.50*** |
|  | (-0.51 - -0.45) | (-0.18 - -0.10) | (-0.26 - -0.19) | (-0.54 - -0.47) | (-0.54 - -0.46) |
| Relative SES^a^ | -1.48*** | -2.16*** | -2.59*** | -1.35*** | -2.41*** |
|  | (-1.56 - -1.41) | (-2.26 - -2.06) | (-2.70 - -2.48) | (-1.43 - -1.27) | (-2.51 - -2.31) |
| SES x (Age – 50) | 0.03** | 0.01 | 0.09*** | 0.05*** | 0.09*** |
|  | (0.01 - 0.04) | (-0.02 - 0.04) | (0.06 - 0.13) | (0.03 - 0.06) | (0.06 - 0.12) |
| SES x (Age – 50)^2^ | 0.07*** | 0.15*** | 0.13*** | 0.07*** | 0.12*** |
|  | (0.06 - 0.08) | (0.13 - 0.16) | (0.11 - 0.14) | (0.06 - 0.08) | (0.11 - 0.14) |
| 1996-99 | *omitted* | *omitted* | *omitted* | *omitted* | *omitted* |
| 2000-03 | -0.04 | -0.03 | -0.00 | 0.04 | -0.06 |
|  | (-0.10 - 0.02) | (-0.10 - 0.05) | (-0.07 - 0.07) | (-0.01 - 0.10) | (-0.12 - 0.01) |
| 2004-07 | 0.09* | 0.09* | 0.14*** | 0.18*** | 0.14*** |
|  | (0.02 - 0.15) | (0.01 - 0.16) | (0.06 - 0.22) | (0.11 - 0.25) | (0.07 - 0.21) |
| 2008-11 | 0.26*** | 0.17*** | 0.18*** | 0.36*** | 0.26*** |
|  | (0.20 - 0.32) | (0.09 - 0.24) | (0.10 - 0.26) | (0.29 - 0.42) | (0.19 - 0.33) |
| 2012-15 | 0.21*** | 0.14*** | 0.16*** | 0.33*** | 0.22*** |
|  | (0.15 - 0.27) | (0.07 - 0.21) | (0.09 - 0.24) | (0.26 - 0.39) | (0.15 - 0.29) |
| 2016-19 | 0.30*** | 0.13** | 0.13** | 0.43*** | 0.22*** |
|  | (0.23 - 0.37) | (0.04 - 0.22) | (0.05 - 0.21) | (0.36 - 0.50) | (0.14 - 0.29) |
| (Age – 50) x |  |  |  |  |  |
| 2000-03 | 0.00 | 0.01 | 0.01 | 0.02* | -0.00 |
|  | (-0.01 - 0.02) | (-0.01 - 0.04) | (-0.01 - 0.03) | (0.00 - 0.03) | (-0.02 - 0.02) |
| 2004-07 | 0.03*** | 0.06*** | 0.04*** | 0.04*** | 0.03** |
|  | (0.01 - 0.05) | (0.03 - 0.08) | (0.02 - 0.07) | (0.02 - 0.06) | (0.01 - 0.05) |
| 2008-11 | 0.03*** | 0.04** | 0.04** | 0.04*** | 0.02* |
|  | (0.01 - 0.04) | (0.02 - 0.06) | (0.01 - 0.06) | (0.02 - 0.06) | (0.00 - 0.05) |
| 2012-15 | 0.04*** | 0.04** | 0.03* | 0.05*** | 0.04*** |
|  | (0.02 - 0.06) | (0.02 - 0.06) | (0.01 - 0.06) | (0.03 - 0.07) | (0.02 - 0.06) |
| 2016-19 | 0.05*** | 0.06*** | 0.03* | 0.07*** | 0.03** |
|  | (0.04 - 0.07) | (0.03 - 0.09) | (0.00 - 0.06) | (0.05 - 0.09) | (0.01 - 0.06) |
| (Age – 50)^2^ x |  |  |  |  |  |
| 2000-03 | -0.01 | -0.01 | -0.01 | -0.01 | -0.00 |
|  | (-0.02 - 0.00) | (-0.02 - 0.00) | (-0.02 - 0.00) | (-0.02 - 0.00) | (-0.01 - 0.01) |
| 2004-07 | -0.01 | -0.01* | -0.02** | -0.01 | -0.01** |
|  | (-0.02 - 0.00) | (-0.03 - -0.00) | (-0.03 - -0.01) | (-0.02 - 0.00) | (-0.02 - -0.00) |
| 2008-11 | -0.02** | -0.02** | -0.02*** | -0.02*** | -0.02** |
|  | (-0.02 - -0.01) | (-0.03 - -0.00) | (-0.03 - -0.01) | (-0.03 - -0.01) | (-0.03 - -0.01) |
| 2012-15 | -0.00 | -0.00 | -0.00 | -0.01 | -0.01 |
|  | (-0.01 - 0.00) | (-0.01 - 0.01) | (-0.02 - 0.01) | (-0.02 - 0.00) | (-0.02 - 0.00) |
| 2016-19 | -0.00 | -0.01 | -0.01 | -0.01 | -0.01 |
|  | (-0.01 - 0.01) | (-0.03 - 0.00) | (-0.03 - 0.00) | (-0.02 - 0.00) | (-0.02 - 0.00) |
| SES x |  |  |  |  |  |
| 2000-03 | 0.04 | 0.01 | 0.14* | -0.02 | 0.16** |
|  | (-0.04 - 0.13) | (-0.11 - 0.14) | (0.01 - 0.26) | (-0.10 - 0.07) | (0.06 - 0.27) |
| 2004-07 | -0.12* | -0.29*** | -0.02 | -0.16** | -0.08 |
|  | (-0.22 - -0.02) | (-0.43 - -0.16) | (-0.16 - 0.12) | (-0.26 - -0.06) | (-0.20 - 0.04) |
| 2008-11 | -0.23*** | -0.43*** | -0.16* | -0.27*** | -0.18** |
|  | (-0.33 - -0.13) | (-0.56 - -0.29) | (-0.30 - -0.02) | (-0.37 - -0.16) | (-0.30 - -0.06) |
| 2012-15 | -0.26*** | -0.51*** | -0.22** | -0.32*** | -0.20** |
|  | (-0.36 - -0.16) | (-0.64 - -0.38) | (-0.36 - -0.09) | (-0.42 - -0.21) | (-0.32 - -0.08) |
| 2016-19 | -0.26*** | -0.47*** | -0.19* | -0.31*** | -0.22*** |
|  | (-0.36 - -0.16) | (-0.62 - -0.31) | (-0.33 - -0.04) | (-0.41 - -0.20) | (-0.35 - -0.09) |
| Constant | -0.41*** | -1.78*** | -1.33*** | -0.66*** | -0.95*** |
|  | (-0.46 - -0.36) | (-1.84 - -1.72) | (-1.39 - -1.27) | (-0.71 - -0.61) | (-1.00 - -0.90) |
| Number of Respondents | 645,946 | 660,730 | 660,356 | 661,979 | 654,321 |

Note: The interactions between period and age (quadratic specification) were jointly significant (p<0.05) for all outcomes.

* p<0.05; ** p<0.01; *** p<0.001

^a^ Rescaled from 0 (bottom percentile) to 1 (top percentile). Because SES was interacted with age and period, the main effect of SES represents the odds ratio for a person aged 50 in the top 1% relative to the bottom 1% of the SES in 1996-99.

Table K. Coefficients (and 95% CIs) from logistic regression models predicting pain outcomes, 1997-2018 NHIS

|  | **Headache** | **Facial/Jaw Pain** | **Neck Pain** | **Low Back Pain** | **Joint Pain** |
| --- | --- | --- | --- | --- | --- |
| Female | 0.95*** | 0.81*** | 0.40*** | 0.22*** | 0.18*** |
|  | (0.93 - 0.97) | (0.78 - 0.85) | (0.38 - 0.42) | (0.20 - 0.23) | (0.17 - 0.20) |
| Age – 50 | -0.22*** | -0.04** | 0.08*** | 0.05*** | 0.30*** |
|  | (-0.24 - -0.21) | (-0.07 - -0.02) | (0.06 - 0.09) | (0.04 - 0.06) | (0.28 - 0.31) |
| (Age – 50)^2^ | -0.08*** | -0.08*** | -0.09*** | -0.07*** | -0.07*** |
|  | (-0.09 - -0.08) | (-0.09 - -0.06) | (-0.10 - -0.08) | (-0.08 - -0.06) | (-0.07 - -0.06) |
| Non-Latinx White | *omitted* | *omitted* | *omitted* | *omitted* | *omitted* |
| Non-Latinx Black | -0.22*** | -0.47*** | -0.43*** | -0.28*** | -0.25*** |
|  | (-0.24 - -0.19) | (-0.52 - -0.42) | (-0.46 - -0.40) | (-0.31 - -0.26) | (-0.27 - -0.22) |
| Non-Latinx Other Race | -0.48*** | -0.65*** | -0.50*** | -0.53*** | -0.72*** |
|  | (-0.53 - -0.44) | (-0.74 - -0.56) | (-0.55 - -0.45) | (-0.57 - -0.49) | (-0.76 - -0.67) |
| Latinx | -0.30*** | -0.38*** | -0.31*** | -0.35*** | -0.58*** |
|  | (-0.32 - -0.27) | (-0.43 - -0.34) | (-0.34 - -0.28) | (-0.38 - -0.33) | (-0.61 - -0.55) |
| Relative SES^a^ | -0.88*** | -0.65*** | -0.74*** | -0.89*** | -0.62*** |
|  | (-0.97 - -0.78) | (-0.79 - -0.52) | (-0.83 - -0.66) | (-0.96 - -0.83) | (-0.70 - -0.54) |
| SES x (Age – 50) | -0.16*** | -0.09*** | -0.11*** | -0.00 | 0.05*** |
|  | (-0.19 - -0.14) | (-0.12 - -0.06) | (-0.12 - -0.09) | (-0.02 - 0.01) | (0.03 - 0.06) |
| SES x (Age – 50)^2^ | -0.01* | 0.04*** | 0.04*** | 0.06*** | 0.04*** |
|  | (-0.03 - -0.00) | (0.03 - 0.06) | (0.03 - 0.05) | (0.05 - 0.06) | (0.03 - 0.05) |
| 1996-99 | *omitted* | *omitted* | *omitted* | *omitted* |  |
| 2000-03 | 0.04 | 0.09 | 0.04 | 0.03 | *omitted* ^b^ |
|  | (-0.03 - 0.11) | (-0.02 - 0.21) | (-0.02 - 0.11) | (-0.02 - 0.08) |  |
| 2004-07 | -0.01 | 0.06 | 0.06 | 0.07* | 0.02 |
|  | (-0.09 - 0.06) | (-0.05 - 0.18) | (-0.01 - 0.12) | (0.01 - 0.12) | (-0.04 - 0.08) |
| 2008-11 | 0.17*** | 0.33*** | 0.23*** | 0.22*** | 0.22*** |
|  | (0.10 - 0.24) | (0.22 - 0.45) | (0.16 - 0.29) | (0.16 - 0.28) | (0.16 - 0.28) |
| 2012-15 | 0.12*** | 0.12* | 0.20*** | 0.25*** | 0.15*** |
|  | (0.05 - 0.19) | (0.01 - 0.24) | (0.13 - 0.27) | (0.20 - 0.31) | (0.09 - 0.22) |
| 2016-19 | 0.20*** | 0.30*** | 0.23*** | 0.25*** | 0.24*** |
|  | (0.12 - 0.28) | (0.18 - 0.43) | (0.15 - 0.31) | (0.18 - 0.31) | (0.16 - 0.31) |
| (Age – 50) x |  |  |  |  |  |
| 2000-03 | 0.00 | -0.02 | 0.02* | 0.01 | ^b^ |
|  | (-0.01 - 0.02) | (-0.05 - 0.01) | (0.00 - 0.04) | (-0.00 - 0.02) |  |
| 2004-07 | -0.01 | -0.00 | 0.04*** | 0.04*** | 0.03*** |
|  | (-0.03 - 0.01) | (-0.04 - 0.03) | (0.02 - 0.06) | (0.03 - 0.05) | (0.01 - 0.05) |
| 2008-11 | -0.01 | -0.01 | 0.03** | 0.04*** | 0.00 |
|  | (-0.04 - 0.01) | (-0.04 - 0.02) | (0.01 - 0.05) | (0.03 - 0.05) | (-0.01 - 0.02) |
| 2012-15 | 0.01 | 0.01 | 0.06*** | 0.06*** | 0.01 |
|  | (-0.01 - 0.03) | (-0.02 - 0.04) | (0.04 - 0.08) | (0.05 - 0.07) | (-0.00 - 0.03) |
| 2016-19 | 0.03** | 0.01 | 0.06*** | 0.07*** | 0.03*** |
|  | (0.01 - 0.05) | (-0.02 - 0.04) | (0.04 - 0.08) | (0.05 - 0.08) | (0.01 - 0.05) |
| (Age – 50)^2^ x |  |  |  |  |  |
| 2000-03 | -0.01 | -0.00 | -0.01 | -0.00 | ^b^ |
|  | (-0.02 - 0.00) | (-0.02 - 0.02) | (-0.02 - 0.00) | (-0.01 - 0.01) |  |
| 2004-07 | -0.01 | -0.00 | -0.01* | -0.00 | -0.01* |
|  | (-0.02 - 0.00) | (-0.02 - 0.01) | (-0.02 - -0.00) | (-0.01 - 0.01) | (-0.02 - -0.00) |
| 2008-11 | -0.02** | -0.01 | -0.02*** | -0.01** | -0.01* |
|  | (-0.03 - -0.01) | (-0.03 - 0.00) | (-0.03 - -0.01) | (-0.02 - -0.00) | (-0.02 - -0.00) |
| 2012-15 | -0.01 | 0.01 | -0.01** | -0.01** | -0.01 |
|  | (-0.02 - 0.00) | (-0.00 - 0.03) | (-0.02 - -0.00) | (-0.02 - -0.00) | (-0.02 - 0.00) |
| 2016-19 | -0.01* | -0.00 | -0.01 | -0.01* | -0.01 |
|  | (-0.02 - -0.00) | (-0.02 - 0.02) | (-0.02 - 0.00) | (-0.02 - -0.00) | (-0.02 - 0.00) |
| SES x |  |  |  |  |  |
| 2000-03 | -0.04 | -0.08 | 0.05 | 0.02 | ^b^ |
|  | (-0.15 - 0.06) | (-0.23 - 0.08) | (-0.04 - 0.14) | (-0.05 - 0.09) |  |
| 2004-07 | -0.07 | -0.10 | -0.05 | -0.12** | -0.08 |
|  | (-0.18 - 0.05) | (-0.28 - 0.08) | (-0.15 - 0.05) | (-0.20 - -0.04) | (-0.18 - 0.02) |
| 2008-11 | -0.17** | -0.35*** | -0.19*** | -0.24*** | -0.21*** |
|  | (-0.28 - -0.06) | (-0.52 - -0.19) | (-0.29 - -0.08) | (-0.33 - -0.15) | (-0.30 - -0.11) |
| 2012-15 | -0.14* | -0.15 | -0.16** | -0.25*** | -0.17*** |
|  | (-0.25 - -0.03) | (-0.33 - 0.02) | (-0.27 - -0.06) | (-0.34 - -0.17) | (-0.26 - -0.07) |
| 2016-19 | -0.17** | -0.32*** | -0.16** | -0.22*** | -0.16** |
|  | (-0.29 - -0.04) | (-0.50 - -0.13) | (-0.28 - -0.05) | (-0.32 - -0.12) | (-0.27 - -0.06) |
| Constant | -1.68*** | -3.02*** | -1.31*** | -0.39*** | -0.24*** |
|  | (-1.75 - -1.62) | (-3.11 - -2.92) | (-1.37 - -1.26) | (-0.44 - -0.35) | (-0.30 - -0.19) |
| Number of  Respondents | 667,955 | 667,900 | 667,918 | 667,903 | 504,194 |

Note: The interactions between period and age (quadratic specification) were jointly significant (p<0.05) for all outcomes except facial/jaw pain.

* p<0.05; ** p<0.01; *** p<0.001

^a^ Rescaled from 0 (bottom percentile) to 1 (top percentile). Because SES was interacted with age and period, the main effect of SES represents the odds ratio for a person aged 50 in the top 1% relative to the bottom 1% of the SES in 1996-99.

^b^ Joint pain was available only since 2002. Therefore, we used 2002-03 as the omitted category.

Fig A. Period Change in Physical Limitations at Age 74 for 10^th^ vs. 90^th^ percentile of SES. These results are based on the models shown in Tables F, H, and J.


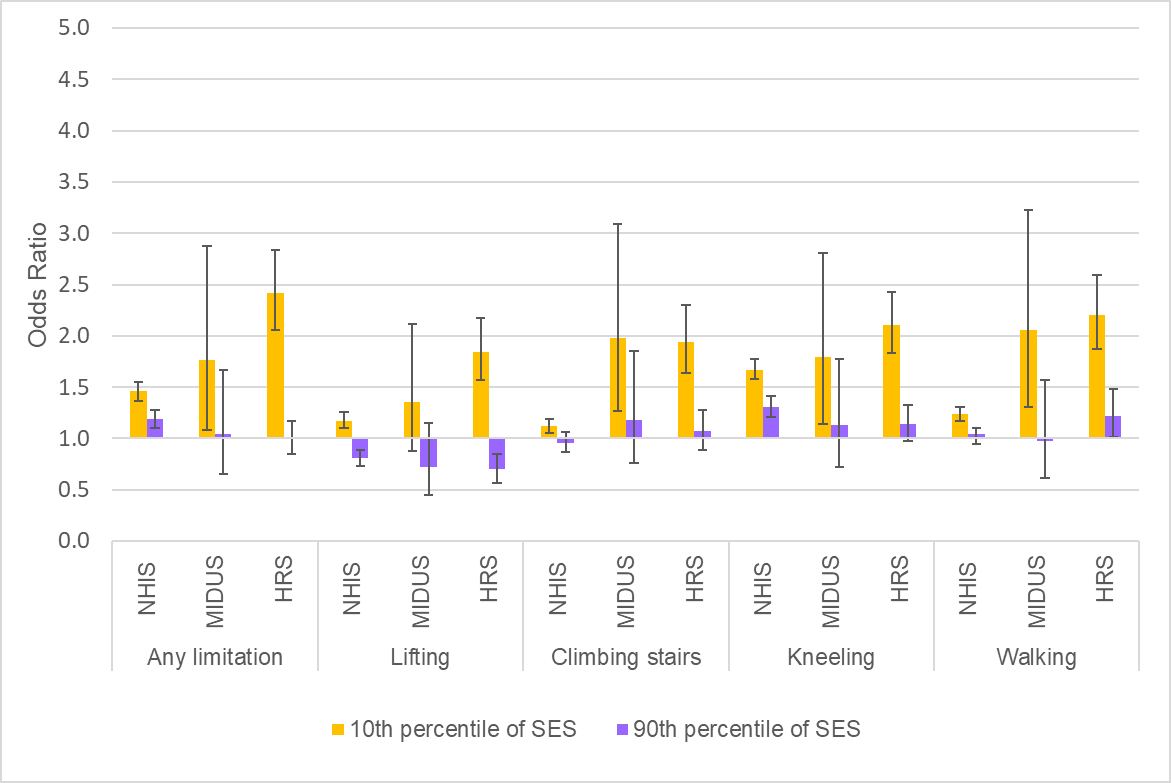


Fig B. Period Change in Overall Pain at age 74 for 10^th^ vs. 90^th^ percentile of SES, HRS 1996-2016.

These results are based on the models shown in Table G.


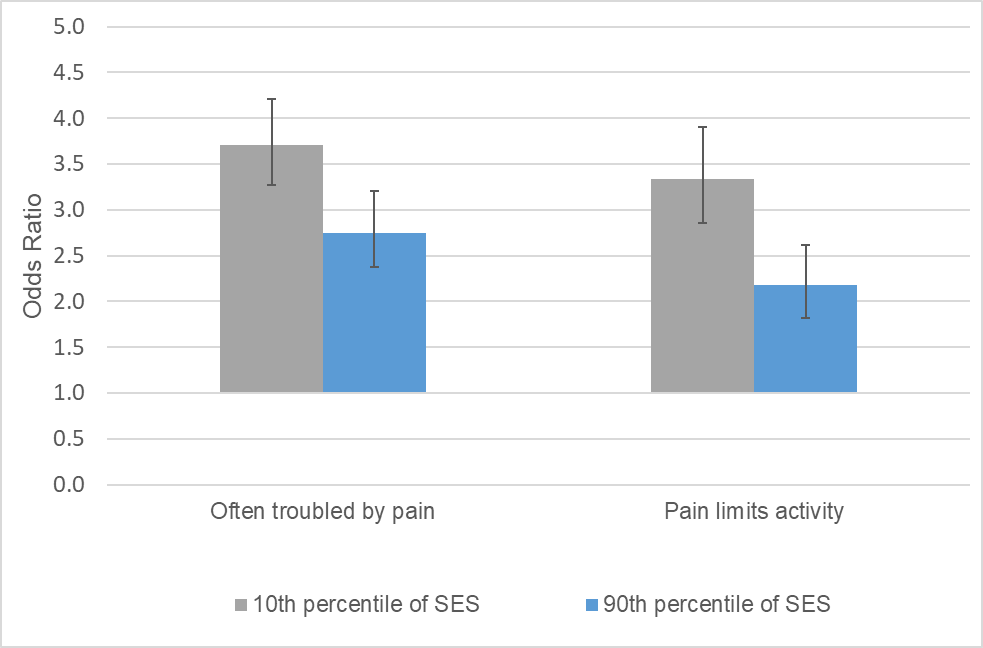


Fig C. Period Change in Specific Types of Pain at Age 74 for 10^th^ vs. 90^th^ percentile of SES. These results are based on the models shown in Tables G, I, and K.


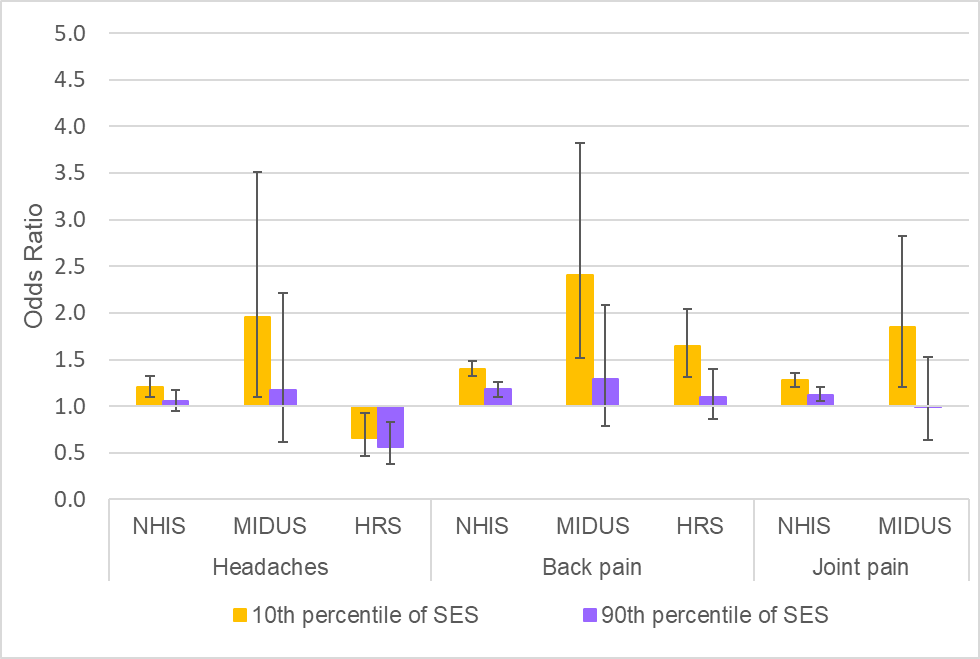


Fig D. Predicted Prevalence of Any Physical Limitation at Age 74 by Survey and Period for Low versus High SES. These predicted probabilities were based on models that adjusted for sex, age (quadratic specification), race/ethnicity, period (categorical specification), and SES. The models included the following 2-way interactions: age (quadratic) x period; age (quadratic) x SES; and period x SES. They also included a 3-way interaction between age (linear), period, and SES. We computed the probabilities for someone aged 74 at the 10th (low SES) vs. the 90th (high SES) percentiles of SES for the specified periods; all other covariates were fixed at the values observed in the sample.


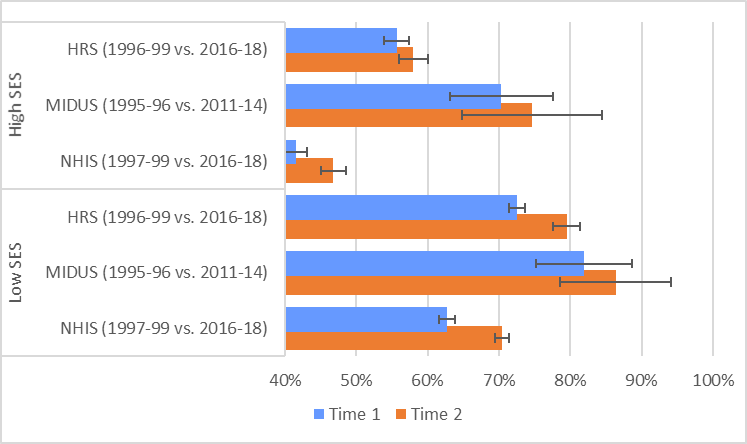


Fig E. Predicted Prevalence of Back Pain at Age 74 by Survey and Period for Low versus High SES. These predicted probabilities were based on models that adjusted for sex, age (quadratic specification), race/ethnicity, period (categorical specification), and SES. The models included the following 2-way interactions: age (quadratic) x period; age (quadratic) x SES; and period x SES. They also included a 3-way interaction between age (linear), period, and SES. We computed the probabilities for someone aged 74 at the 10th (low SES) vs. the 90th (high SES) percentiles of SES for the specified periods; all other covariates were fixed at the values observed in the sample.


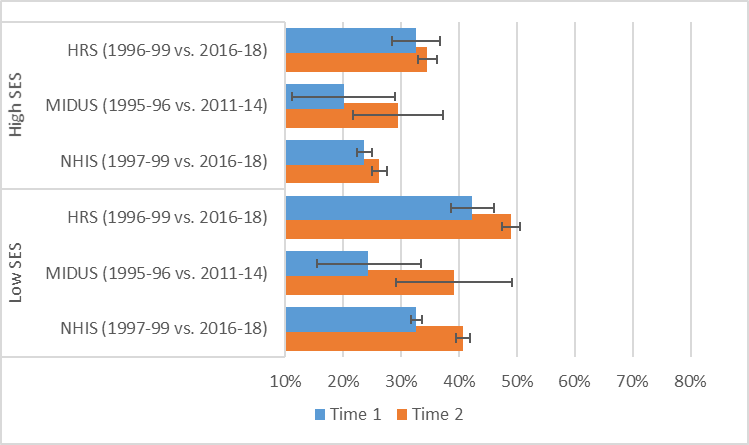


# References

1. Goldman N, Glei DA, Weinstein M. Declining mental health among disadvantaged Americans. ProcNatlAcadSciUSA. 2018;115(28):7290–5.

2. Glei DA, Goldman N, Weinstein M. Perception has its own reality:  subjective versus objective measures of economic distress. Popul Dev Rev. 2018;44(4):695–722.

3. Glei DA, Stokes AC, Weinstein M. Widening socioeconomic disparities in pain and physical function among Americans are linked with growing obesity. Journal of Aging and Health [Internet]. 2021;28(08982643211028121). Available from: https://doi.org/10.1177%2F08982643211028121

4. Glei DA, Lee C, Weinstein M. Socioeconomic disparities in U.S. mortality: The role of smoking and alcohol/drug abuse. SSM - Population Health. 2020 Dec 1;12:100699.

5. Glei DA, Stokes A, Weinstein M. Changes in mental health, pain, and drug misuse since the mid-1990s: Is there a link? Soc Sci Med. 2020;246:112789.

6. Glei DA, Weinstein M. Mental health, pain, and risk of drug misuse: A nationwide cohort study. Addict Behav. 2020 Oct;109:106467.

7. Glei DA, Weinstein M. Drug and alcohol abuse: the role of economic insecurity. AmJHealth Behav. 2019;43(4):838–53.

8. RAND Center for the Study of Aging. RAND HRS Longitudinal File 2018 (V1) Documentation, includes 1992-2018 (early release) [Internet]. 2021. Available from: https://hrsdata.isr.umich.edu/sites/default/files/documentation/other/1615843861/randhrs1992_2018v1.pdf

9. von Hippel PT, Scarpino SV, Holas I. Robust estimation of inequality from binned incomes. Sociological Methodology. 2016;46(1):212–51.

10. National Center for Health Statistics. Multiple imputation of family income and personal earnings in the National Health Interview Survey: methods and examples [Internet]. U.S. Department of Health and Human Services, Centers for Disease Control and Prevention, National Center for Health Statistics, Divions of Health Interview Statistics; 2019. Available from: https://www.cdc.gov/nchs/data/nhis/tecdoc18.pdf

11. StataCorp. Stata: Release 16. Statistical Software. College Station, TX: StataCorp LLC; 2019.

12. Rubin DB. Multiple imputation after 18+ years (with discussion). J Am Stat Assoc. 1996;91(Journal Article):473–89.

13. Schafer JL. Multiple imputation: a primer. StatMethods MedRes. 1999;8(1):3–15.

14. Royston P, Carlin JB, White IR. Multiple imputation of missing values: new features for mim. Stata Journal. 2009;9(2):252–64.

15. Brim OG, Baltes PB, Bumpass LL, Cleary PD, Featherman DL, Hazzard WR, et al. National Survey of Midlife Development in the United States (MIDUS), 1995-1996.  Technical Report on Methodology. Ann Arbor, MI: Inter-university Consortium for Political and Social Research; 2016.

16. Palit CD, Radler B, Lein V. Midlife in the United States (MIDUS Refresher), 2011-2014: MIDUS refresher sampling and weighting. Inter-university Consortium for Political and Social Research [distributor]; 2016.

17. National Center for Health Statistics. Survey description, National Health Interview Survey, 2018 [Internet]. Hyattsville, MD: National Center for Health Statistics; 2019 [cited 2021 Jun 8]. Available from: https://ftp.cdc.gov/pub/Health_Statistics/NCHS/Dataset_Documentation/NHIS/2018/srvydesc.pdf

18. Sonnega A, Faul JD, Ofstedal MB, Langa KM, Phillips JW, Weir DR. Cohort Profile: the Health and Retirement Study (HRS). IntJEpidemiol. 2014;43(2):576–85.

19. HRS Staff. Sample Sizes and Response Rates, Health and Retirement Study [Internet]. Ann Arbor, MI: Institute for Social Research, University of Michigan; 2017. Available from: https://hrs.isr.umich.edu/publications/biblio/9042
