# Supplementary material for: Disadvantaged Americans are suffering the brunt of rising pain and physical limitations
Source: PLoS One. 2021 Dec 9;16(12):e0261375. doi: 10.1371/journal.pone.0261375 (PMC8659634; doi:10.1371/journal.pone.0261375)
Supplement: Attachment — Submitted filename: Response-to-Reviewers-2021-10-26.docx [file pone.0261375.s002.docx]

# Reviewer 1

*Thanks for the opportunity to review the manuscript “Disadvantaged Americans are suffering the brunt of rising pain and physical limitations” (PONE-D-21-24383). The paper utilizes three national datasets to examine the disparities in the development of pain and physical limitation for people in different SES groups. The research design is solid, and the findings are important. I only have several suggestions about the clarification of research goal and method. I raise them below in no particular order.*

*1) In the introduction part: though I appreciate that the authors stated their hypotheses clearly, it seems a little odd to see hypotheses pop out suddenly without a clear and organized literature review. For example, on the second and third paragraph (line 48 and 56 respectively), hypotheses are mentioned at the beginning of the paragraph before solid evidence from previous literature is discussed.*

**Response:** We moved the first sentence of the second paragraph of the Introduction to the end of the fourth paragraph. Then, we inserted a topic sentence in the second paragraph that introduces the evidence regarding age variation in the trends.

In addition, we moved the first sentence of the third paragraph of the Introduction to the end of the fifth paragraph.

*2) The biggest innovation of the paper is use multidimensional measure of SES, but the innovation and advantages of this measure over other commonly used measures are yet clearly discussed. I suggest use a separate, independent paragraph for this.*

**Response:** As suggested by the reviewer, we have added a paragraph in the Introduction that highlights the advantages of using a multidimensional measures of SES. In S1 Appendix, we also added references to the previous papers that have used a similar measure.

*3) The aim of using age and period interactions are not clear enough to me. For line 164-165, why including age and period interactions can test “whether rising pain and growing physical limitations are concentrated in midlife”? Also, why only 50-year-old is used to show the period effects given that all three datasets covered 50 and other above 50 aged groups? What if another age is used? This could be added in the sensitivity analysis.*

**Response:** As noted in the Introduction, the previous literature led us to suspect that the rise in pain and physical limitations would be more concentrated in midlife. Therefore, we included interactions between age and the period effect in order to test whether the period trend in pain/physical limitations differed significantly by age. In these models (S6-S11 Tables), the main effect for period represents the change over time in pain/physical limitations for someone aged 50 (because we centered age at 50) and the main effect of age (quadratic specification) represents the age pattern of pain/physical limitations in the omitted (i.e., reference) period. The interactions between period and age indicate the extent to which change over time differs by age. In many cases (particularly for NHIS and HRS where we have more statistical power), the age-by-period interaction terms were jointly significant, which suggests that the trends did, in fact, vary by age. [See footnotes we added to S6-S11 Tables.] Thus, the model would be misspecified if those interactions were omitted.

For Figures 3-5, we showed the period change at age 50 for the 10^th^ and 90^th^ percentiles of SES. It is true that we could have shown those period changes for any age included in all three surveys (50-74). As suggested by the reviewer, we have added similar figures for age 74 in the Supplementary Material to capture the full age range covered by all three surveys. The period increase in various types of physical limitations, any pain, and activity limiting pain tended to be greater at age 74 (S1 & S2 Figures) than at age 50 (Fig 3 & 4), but the patterns by SES were similar at both ages. Figure 5 (for age 50) and S3 Figure (for age 74) also consistently showed an increase over time in back and joint pain among those with low SES. In contrast, the results were mixed for those with high SES: HRS indicated no significant increase in back pain at either age 50 or 74; NHIS & MIDUS showed an increase in back and joint pain at age 50 (albeit much smaller than for those with low SES), but at age 74, only NHIS indicates a significant increase in back and joint pain. We have modified that part of the Results section to note the differences in the results at age 50 vs. 74. [Note: Between ages 25 and 49, we could show similar graphs only for NHIS and MIDUS (because HRS did not target the population younger than 50). At ages 75 and older, we could show similar graphs only for NHIS and HRS (because MIDUS did not target the population older than 74). However, there is no need to show a graph for every possible age because S6-S11 Tables provide the full results from all models. The reader could compute the corresponding odds ratios for any age.]

Figures 6-8 show the period effects based on the models fit separately to three age groups. Those graphs also show how the period effects vary between ages 25-49, 50-74, and 75+.

Figures 9 and 10 show the predicted prevalence of any limitation and back pain at age 50 by survey and period for low vs. high SES. For completeness, we have also added corresponding graphs for age 74 in the Supplementary Material (S4 & S5 Figures).

*4) Line 169: why HRS data are the only unweighted here?*

**Response:** Unfortunately, Stata 16.1 would not allow us to use weights that vary over time for the same individual in a multilevel logit model with a random individual-level intercept. If we were estimating a linear model, we could use the Huber-White estimator to correct the standard errors for individual-level clustering rather than using a multilevel model. However, that solution is less than ideal for two reasons: a) it would produce different results in the case of a non-linear model (i.e., represents the population average effect rather than the subject specific effect^[1]^); and b) we would be unable to correct for PSU-level clustering (as we do in the random intercept model). Thus, given the choice between correcting for intra-individual clustering and using weighted regression, we opted for the former.

Some statisticians would argue that one should always use unweighted regression because any covariates that are important should be included in the model. As a test of sensitivity, we refitted the models for MIDUS and NHIS using unweighted regression. When the data from all three surveys were modeled using unweighted data, the estimates of SES widening based on NHIS remained similar, but the estimates from MIDUS were somewhat smaller (e.g., OR=1.55, 95% CI 1.10-2.17 for any physical limitation compared with 1.70, 95% CI 1.15-2.51 in Figure 1). We have no way of knowing whether one set of estimates is more *accurate* than the other. While the estimates for MIDUS changed somewhat depending on whether or not the regression was weighted, the pattern of the results remained unchanged: the SES disparity widened significantly because pain/limitations increased more for those with low SES than for those with high SES.

In the Modeling Strategy section, we have added information about the sensitivity of the results to weighting.

INTERNAL NOTES (will be hidden in the submission): Because HRS is longitudinal dataset with up to 11 observations per respondent, it is important to include a random intercept to allow for the fact that repeated observations for the same respondent over time are likely to be correlated (i.e., intra-individual clustering). In HRS, the survey weights vary over time for a given respondent (because the weights are intended to represent the US population in the relevant age range at the time of that survey wave). Unfortunately, we have been unable to fit a random intercept logit model using survey weights that vary over time for the same individual. In Stata 16.1, the “xtlogit” command does not allow weights that vary over time. Although the manual indicates that the “melogit” command will allow weights that vary over time, we have been unable to get the models to run (i.e., Stata returns an error message “initial values not feasible”). We also tried using the user-written “gllamm” command, but a single model ran for 50 minutes without converging and most of the iterations were “not concave.”

1) How much did the weights change over time for a given individual?

- It looks like the weights are designed to represent the total population at that wave (e.g., the sample size is similar in 1998 and 2016, but the sum of the weights are 68K in 1998 vs. 107K in 2016; the mean of the weights in each wave ranges from 2751 in 1995/96 to 5533 in 2016).
- Thus, I rescaled the weights for each wave so that they sum to the unweighted N for the wave (i.e., average of the weights for that wave is 1.0;). Then, I checked the variation in the rescaled weights.
- There is quite a lot of variation in the weights over time for the same individual. I computed the difference between the min and the max weights for each R. The mean difference was 0.3, but nearly 5% of the Rs exhibited a difference greater than 1.0. The ratio between the max/min weight was 1.47, but nearly 10% of Rs had a ratio greater than 2.0.
  - For the R with the biggest differential in weights over followup (HHIDPN=123785010), the rescaled weight was 3.52 in 1998, but ranged from 1.92 (in 2016) to 7.75 (in 2000).
  - For the R with the biggest ratio of max/min weights (HHIDPN=502553021), the rescaled weight was 2.74 in 2006 (which was the maximum across all available waves), but the weights for 2010-2016 ranged from 0.082 to 0.097.

2) How much of a difference does it make if we run regression models using the rescaled weights fixed at the 1^st^ wave for R but no correction for PSU clustering?

- Any physical limitation: Based on the weighted model, the change in the SES disparity was even greater (OR=2.79 vs. 2.43 in Fig 1). At age 50, the period change was OR=1.09 (NS) for the 10^th^ %ile and OR=0.39*** for the 90^th^ %ile (vs. 1.26+ and 0.52***, respectively, in Fig 3). At age 74, the period change was OR=2.63*** for the 10^th^ %ile and OR=0.95 (NS) for the 90^th^ %ile (vs. 2.42*** and 1.00 (NS), respectively, in S1 Fig).
- Backpain: Based on the weighted model, the change in the SES disparity was even greater (OR=1.57*** vs. 1.49*** in Fig 1). At age 50, the period change was OR=1.27* for the 10^th^ %ile and OR=0.81* for the 90^th^ %ile (vs. 1.52** and 1.02 (NS), respectively, in Fig 3). At age 74, the period change was OR=1.57*** for the 10^th^ %ile and OR=1,99 (NS) for the 90^th^ %ile (vs. 1.64*** and 1.10 (NS), respectively, in S1 Fig). For this outcome, the effect size for SES widening is somewhat bigger when using weighted data, but the period changes at different levels of SES are slightly larger.

# Reviewer 2

*In this well-written paper, the authors pursue an important research question carefully and rigorously: Have socioeconomic disparities in pain and physical limitations widened over time in the U.S., and does the answer to this question depend on the age group one is examining? A small but growing number of recent articles have examined temporal trends in pain and pain disparities, but this one directly tackles the interaction between disparities and age group, and carefully examines whether changes in disparities are driven by improving or declining health in each age group.

The authors use 3 different large, national data sets; smart analytic choices; and a number of important robustness checks to answer their research questions with high rigor. For example, by using a relative measure of SES (90th vs. 10th percentile), they circumvent the possibility of lagged selection bias that has plagued some recent work on changes in disparities. The detailed supplementary materials further underscore the authors’ thorough knowledge of their data, variables, and findings. The main weaknesses are relatively minor, and have to do with conceptualization and interpretration; e.g., a lack of clarity about the relationship between pain and functional limitations.*

## MAJOR COMMENTS

*Results: Different age groups often appear to have overlapping confidence intervals in the figures. For example, in Figures 6-8, the CIs for HRS respondents ages 50-74 and 75+ nearly always overlap (although the point estimates are consistently higher for the 50-74 group). Don’t these overlapping CIs weaken the authors’ claim that SES disparities have widened more for the younger group?*

**Response:** Overlapping CIs across subgroups do not necessarily indicate that the subgroup is not significant (although the converse is true: If the CIs do not overlap, the subgroup difference will be significant). A difference between subgroups can be significant even if the CIs overlap (see <https://towardsdatascience.com/why-overlapping-confidence-intervals-mean-nothing-about-statistical-significance-48360559900a>; this PhD student did a better job of explaining it than I can).

As noted in the Modeling Strategy, we formally tested whether SES widening varied significantly by age by adding a three-way interaction among age, SES, and period to the models based on the pooled data (all ages available for each survey). Those tests demonstrated that SES disparities in physical limitations and overall pain widened more at younger ages than at older ages, which was consistent with the patterns observed in the models stratified by age group. We have added a sentence in the results section to emphasize that point.

*Discussion: The latter, speculative half of the Discussion is weaker than earlier (more methodological or descriptive) parts of the text, with several unclear claims. One reason is because the authors are never explicit (in the Discussion or elsewhere) of what they see as the link between pain and functional limitations. They seem to presume a close link between the two--hence they can describe “the contradictory increase in pain but improvement in physical function among those with high SES”--but how close? Functional limitations could reflect problems with fatigue, balance/coordination, cognitive function, etc. as well as problems with pain. Couldn’t pain and function show different patterns because they are reflecting different underlying problems? This should be spelled out. (Additional, more minor comments about the Discussion are below.)*

**Response:** Numerous studies have established a strong association between pain, functional limitations, physical performance, and ADL disability (Andrews et al., 2013; Azevedo et al., 2012; Covinsky et al., 2009; Di Iorio et al., 2007; Edmond & Felson, 2003; Leveille et al., 2007; Reid et al., 2005; Scudds & Østbye, 2001). Covinsky (2009) reported that individuals who reported significant pain exhibited a degree of functional limitation comparable with their pain-free counterparts who were two to three decades older. They (2009, p. 1558-1559) argue that the relationship between pain and physical function is likely to be bidirectional and may involve “mutual feedback loops in which pain and functional limitations are mutually reinforcing, with pain exacerbating functional limitations and functional limitations exacerbating pain…The powerful and robust association between pain and functional limitations suggests that pain and functional limitations may often be part of the same underlying pathophysiological process.” Similarly, Andrews et al. (2013, p. 583) concludes that, “Like many geriatric syndromes, pain and disability may represent interrelated phenomena that occur simultaneously.”

The presumed link between pain and physical function is so widely accepted that the National Pain Strategy proposed a new construct that combines them: high-impact chronic pain is defined as persistent pain with substantial restriction of life activities for six months or more (Dahlhamer, 2018; Interagency Pain Research Coordinating Committee, 2016; Von Korff et al., 2016).

Given the well-established correlation between pain and physical function, we expected that an increase in pain over time at the population-level would be accompanied by a corresponding decline in physical function. We have added a paragraph in the Discussion noting: 1) the prior evidence regarding the close link between pain and physical function; 2) our expectation that trends in pain and physical would be similar; and 3) the apparent contradictory results for physical limitations and overall pain among those with high SES.

## MINOR COMMENTS

*Abstract: I suggest specifying the time period under examination early in the abstract.*

**Response:** In principle, that is a good idea. However, doing so is complicated by the fact that we use three datasets, each of which covers a different time period (i.e., 1997-2018 for NHIS, 1995-96 vs. 2011-14 for MIDUS; 1996-2016 for HRS). Given that the abstract is limited to 300 words, we could not devote the necessary space to spell out the survey name and time period covered by each dataset. Thus, we have added the following clause in the first sentence of the Abstract: “covering the period from the mid-1990s to the mid-2010s.”

*First page of Introduction (two questions): (1) “least able to bear the consequences” is vague. What kinds of consequences?*

Response: We have removed that clause to avoid confusion. [FYI: That clause was intended to refer to the consequences of rising pain and growing physical limitations mentioned earlier in that same paragraph (i.e., diminished quality of life, higher health care costs, reduced earning capacity, reliance on disability benefits, and greater need for long-term care).]

*(2) Authors write, “We hypothesize that the gap in SES disparities has widened more at younger ages (25-49) than among the oldest Americans.” However, elsewhere in the paper, the focus is on “midlife” adults (50-74) vs. older ones (75+), not “younger” adults. E.g., the abstract tells us that disparities “widened even more in midlife than in late life”. For consistency, the authors may wish to clarify that they focus on *3* different age groups, and that they hypothesize and find that disparities are typically larger among younger than older age groups. (Even removing the “(25-49)” from the quoted sentence would help, since then readers aren’t primed to focus specifically on that group vs. all others.)*

**Response:** Thank you for pointing out this inconsistency. In response to comments from Reviewer #1, we have moved the sentence in question to the end of the fifth paragraph of the Introduction (where it now closely follows the sentence defining the three age groups used in our analysis). In addition, as suggested by the reviewer, we have revised the sentence to read, “We hypothesized that the gap in SES disparities widened more at younger ages than among older Americans.”

*Discussion: the authors imply that “perceived pain” is somehow different from “real pain”--but *all* pain is “perceived pain”. One could fairly ask whether pain *reporting* styles have changed over time, but to imply that pain exacerbated by opioid-related sensitization is less “real” than other pain is confusing.*

**Response:** Yes, all pain is “perceived pain”, however we recognize that other factors can influence a person’s perception of pain. For example, prolonged use of opioids can cause changes in the brain that increase sensitivity to pain and desensitize the dopamine system to naturally-rewarding experiences (Ballantyne & Mao, 2003; Volkow et al., 2011). Thus, it is possible that aggressive marketing and over-prescribing of opioids has contributed to opioid-induced hyperalgesia, which in turn amplifies pain perception. Even the experience of prolonged chronic pain affects perceptions. Garland et al. (2013) suggest that recurrent pain initiates a downward spiral in which the individual's attention becomes fixated on the pain, thus exacerbating the perception of pain and fueling negative affect. This maladaptive cognitive response to chronic pain is also referred to as “pain catastrophizing” (Institute of Medicine, 2011).

We have no intention of implying that opioid-induced hyperalgesia is somehow “less real.” However, the fact that pain is inherently subjective means that it is susceptible to many influences that alter perceptions. That doesn’t necessarily mean people have changed their pain reporting style over time, it could simply reflect changes in those influences. In any case, we have removed references to “perceived” in order to avoid implying that there is somehow a difference between perceived pain and “real” pain.

The key point we are trying to make here is that even if opioid use (or some other influence) may explain part of the rise in pain, it would not explain the apparent contradiction among those with high SES: overall pain increased whereas there was little change (and perhaps even improvement) in physical function.

*Discussion: The quote from Joffe-Walt is intriguing, but also a bit of a non-sequitur in context. I was not clear on its implications in the authors’ view. Is the idea that low-SES individuals are exaggerating/fabricating their degree of functional limitation, in order to quality for disability benefits?*

**Response:** We have eliminated those two sentences.

# References

Andrews, J. S., Cenzer, I. S., Yelin, E., & Covinsky, K. E. (2013). Pain as a Risk Factor for Disability or Death. *Journal of the American Geriatrics Society*, *61*(4), 583–589. https://doi.org/10.1111/jgs.12172

Azevedo, L. F., Costa-Pereira, A., Mendonça, L., Dias, C. C., & Castro-Lopes, J. M. (2012). Epidemiology of chronic pain: A population-based nationwide study on its prevalence, characteristics and associated disability in Portugal. *The Journal of Pain*, *13*(8), 773–783. https://doi.org/10.1016/j.jpain.2012.05.012

Ballantyne, J. C., & Mao, J. (2003). Opioid therapy for chronic pain. *The New England Journal of Medicine*, *349*(20), 1943–1953. https://doi.org/10.1056/NEJMra025411

Covinsky, K. E., Lindquist, K., Dunlop, D. D., & Yelin, E. (2009). Pain, functional limitations, and aging. *Journal of the American Geriatrics Society*, *57*(9), 1556–1561. https://doi.org/10.1111/j.1532-5415.2009.02388.x

Dahlhamer, J. (2018). Prevalence of Chronic Pain and High-Impact Chronic Pain Among Adults—United States, 2016. *MMWR. Morbidity and Mortality Weekly Report*, *67*. https://doi.org/10.15585/mmwr.mm6736a2

Di Iorio, A., Abate, M., Guralnik, J. M., Bandinelli, S., Cecchi, F., Cherubini, A., Corsonello, A., Foschini, N., Guglielmi, M., Lauretani, F., Volpato, S., Abate, G., & Ferrucci, L. (2007). From Chronic Low Back Pain to Disability, a Multifactorial Mediated Pathway. *Spine*, *32*(26), E809–E815. https://doi.org/10.1097/BRS.0b013e31815cd422

Edmond, S. L., & Felson, D. T. (2003). Function and Back Symptoms in Older Adults. *Journal of the American Geriatrics Society*, *51*(12), 1702–1709. https://doi.org/10.1046/j.1532-5415.2003.51553.x

Garland, E. L., Froeliger, B., Zeidan, F., Partin, K., & Howard, M. O. (2013). The downward spiral of chronic pain, prescription opioid misuse, and addiction: Cognitive, affective, and neuropsychopharmacologic pathways. *Neuroscience and Biobehavioral Reviews*, *37*(10 Pt 2), 2597–2607. https://doi.org/10.1016/j.neubiorev.2013.08.006

Institute of Medicine. (2011). *Relieving pain in America: A blueprint for transforming prevention, care, education, and research*. National Academies Press. https://doi.org/10.17226/13172

Interagency Pain Research Coordinating Committee. (2016). *National Pain Strategy—A Comprehensive Population Health -Level Strategy for Pain* (p. 84). US Department of Health and Human Services, National Institutes of Health.

Leveille, S. G., Bean, J., Ngo, L., McMullen, W., & Guralnik, J. M. (2007). The pathway from musculoskeletal pain to mobility difficulty in older disabled women. *Pain*, *128*(1–2), 69–77. https://doi.org/10.1016/j.pain.2006.08.031

Reid, M. C., Williams, C. S., & Gill, T. M. (2005). Back pain and decline in lower extremity physical function among community-dwelling older persons. *The Journals of Gerontology. Series A, Biological Sciences and Medical Sciences*, *60*(6), 793–797. https://doi.org/10.1093/gerona/60.6.793

Scudds, R. J., & Østbye, T. (2001). Pain and pain-related interference with function in older Canadians: The Canadian study of health and aging. *Disability and Rehabilitation*, *23*(15), 654–664. https://doi.org/10.1080/09638280110043942

Volkow, N. D., Wang, G. J., Fowler, J. S., Tomasi, D., & Telang, F. (2011). Addiction: Beyond dopamine reward circuitry. *Proceedings of the National Academy of Sciences of the United States of America*, *108*(37), 15037–15042. https://doi.org/10.1073/pnas.1010654108

Von Korff, M., Scher, A. I., Helmick, C., Carter-Pokras, O., Dodick, D. W., Goulet, J., Hamill-Ruth, R., LeResche, L., Porter, L., Tait, R., Terman, G., Veasley, C., & Mackey, S. (2016). United States National Pain Strategy for Population Research: Concepts, Definitions, and Pilot Data. *The Journal of Pain*, *17*(10), 1068–1080. https://doi.org/10.1016/j.jpain.2016.06.009

1. As Germán Rodríguez explains (<https://data.princeton.edu/pop510/hospmle>), the population average effect is obtained by “integrating out” the random effect. In contrast, the effects from the random effect model represent a subject specific effect (i.e., conditional effect given a fixed value of the random effect). See also: <https://www.stata.com/support/faqs/statistics/random-effects-versus-population-averaged/>. For a linear model, it turns out that the population-average effect and the subject specific effect are the same.
